# Distinct N-terminal regions of the exomer secretory vesicle cargo Chs3 regulate its trafficking itinerary

**DOI:** 10.3389/fcell.2014.00047

**Published:** 2014-09-03

**Authors:** Amanda M. Weiskoff, J. Christopher Fromme

**Affiliations:** Department of Molecular Biology and Genetics, Weill Institute for Cell and Molecular Biology, Cornell UniversityIthaca, NY, USA

**Keywords:** cargo sorting, Golgi, secretory vesicle, AP-1 complex, chitin synthase

## Abstract

Cells transport integral membrane proteins between organelles by sorting them into vesicles. Cargo adaptors act to recognize sorting signals in transmembrane cargos and to interact with coat complexes that aid in vesicle biogenesis. No coat proteins have yet been identified that generate secretory vesicles from the *trans*-Golgi network (TGN) to the plasma membrane, but the exomer complex has been identified as a cargo adaptor complex that mediates transport of several proteins in this pathway. Chs3, the most well-studied exomer cargo, cycles between the TGN and the plasma membrane in synchrony with the cell cycle, providing an opportunity to study regulation of proteins that cycle in response to signaling. Here we show that different segments of the Chs3 N-terminus mediate distinct trafficking steps. Residues 10–27, known to mediate retention, also appear to play a role in internalization. Residues 28–52 are involved in transport to the plasma membrane and recycling out of endosomes to prevent degradation in the vacuole. We also present the crystal structure of residues 10–27 bound to the exomer complex, suggesting different cargo adaptors could compete for binding to this segment, providing a potential mechanism for regulation.

## Introduction

Eukaryotic cells must transport transmembrane proteins between different subcellular compartments, often in response to specific signals or conditions. This transport is mediated by coat complexes, which help to form the shape of transport vesicles (Bonifacino and Glick, 2004). These coat complexes also contain or interact with adaptor proteins that recognize sorting signals in the cytosolic domains of cargo proteins to sort them into the vesicles.

No coat complexes are known to mediate transport directly from the *trans*-Golgi network (TGN) to the apical plasma membrane (PM) of polarized cells, which corresponds to secretory vesicles in the model organism *Saccharomyces cerevisiae* (budding yeast). Therefore, it remains poorly understood how cells regulate this trafficking step. One of the many transmembrane proteins that follow this route of transport in yeast is the chitin synthase enzyme Chs3. Chs3 cycles between the TGN and the cell surface in a cell cycle dependent manner (Chuang, 1996; Ziman et al., 1998; Zanolari et al., 2011). This localization pattern is reminiscent of other proteins for which localization is regulated by signaling, such as the human Glut4 glucose transporter (Bryant et al., 2002). Chs3 is localized to the bud neck (junction of mother and daughter cells) through its interaction with its activator Chs4, which binds the septin-interacting protein Bni4 (DeMarini, 1997; Reyes et al., 2007).

The transport of Chs3 to the cell surface requires the exomer complex, which acts as a cargo adaptor for Chs3 and other cargos. Exomer consists of the core protein Chs5, and four paralagous adaptor proteins called Chs5-Arf1-binding Proteins (ChAPs): Chs6, Bud7, Bch1, and Bch2. Deletion of the *CHS5* or *CHS6* genes, or simultaneous deletion of the *BUD7* and *BCH1* genes, prevents Chs3 transport to the cell surface (Santos and Snyder, 1997; Sanchatjate and Schekman, 2006; Trautwein et al., 2006; Wang et al., 2006; Starr et al., 2012).

Two other proteins have been identified as exomer cargos: Fus1, a protein involved in mating, and Pin2, a prion-like domain-containing protein. Transport of Pin2, unlike Chs3, requires Bch1 or Bch2, and requires the C-terminus of Pin2, which has little similarity to any part of Chs3 (Ritz et al., 2014) Fus1 has a sorting signal not found in Chs3 and requires Bch1 and Bud7 for transport (Barfield et al., 2009). This suggests that exomer recognizes multiple motifs, possibly through interaction with different subsets of ChAPs.

Retention of Chs3 at the TGN requires AP-1, an adaptor protein complex that mediates trafficking between the TGN and endosomes (Valdivia et al., 2002). Disruption of the AP-1 complex partially rescues the phenotype of an exomer deletion, due to escape of Chs3 to the cell surface when both exomer and AP-1 pathways are blocked. Disruption of the Gga1/2 clathrin adaptors has a similar effect (Copic et al., 2007).

It was recently reported that both the exomer dependent transport and the AP-1 dependent retention of Chs3 are mediated by a motif near the N-terminus of the protein, _19_DEESLL_24_ (Starr et al., 2012). An interaction between exomer and the C-terminus of Chs3 was also found to be required for its transport out of the TGN (Rockenbauch et al., 2012).

In this study we examine the interaction between Chs3 and exomer. We find that different regions of Chs3 play different roles in balancing Chs3 traffic to and away from the PM. We also present a crystal structure of a portion of the Chs3 N-terminus bound to the exomer complex. We propose a role for the N-terminus in regulating both transport and retention of Chs3 by facilitating competition between the protein complexes required for these two processes.

## Materials and methods

### Microscopy

Cells were grown to log phase (OD_600_ ~ 0.5) in synthetic dropout media, and imaged on a DeltaVision RT wide-field deconvolution microscope (Applied Precision). Images were deconvolved in SoftWoRx 3.5.0 software (Applied Precision) and min/max light levels adjusted for clarity in ImageJ (Abramoff et al., 2004) with levels kept consistent within each experiment.

### Exomer purification

Recombinant “core” exomer complex (Chs5 residues 1–77 and Chs6-6xHis) was purified as described for the Chs5(1–299)/Chs6-6xHis construct (Paczkowski et al., 2012). Protein was concentrated to ~25 mg/ml for crystallography, or 5 mg/ml for the interaction assay.

### Interaction assay

GST-Chs3 fragment constructs were constructed in the pGEX-2T vector (GE Healthcare) and transformed into Rosetta2 (DE3) *E. coli* cells (Novagen) for expression. 1 L culture was grown to ~3 OD_600_ in TB media at 37°C, temperature lowered to 18°C, then expression induced with 240 uM IPTG. After overnight expression, cells were harvested by centrifugation, resuspended in 50 ml PBS buffer with 1 mM DTT, and lysed by sonication. GST fusion proteins were isolated by adding 100 μl equilibrated glutathione resin (G-Biosciences) to 5 ml cleared lysate and incubating with rotation at least 2 h at 4°C. Resin was washed 3 times with 1 ml PBS+DTT and resuspended in 500 μl PBS+DTT. 10 μl of 5 mg/ml exomer protein was added and mixture was incubated ~1 h at 4°C. Resin was washed 3 times with 1 ml PBS+DTT and analyzed by SDS-PAGE and Western blot with anti-6xHis antibody (Covance).

### Crystallography

The Chs5(1–77)/Chs6-6xHis exomer complex was co-crystallized with Chs3 peptides (Genscript, 95% purity) using the hanging drop vapor diffusion method. The peptides were resuspended in the precipitant solution (0.3 M ammonium sulfate, 0.1 M citric acid pH 4.0) to a concentration of 100 μ M. 1 μl of the peptide solution was mixed with 1 μl of 25 mg/ml exomer, resulting in a molar ratio of 3.85:1 peptide:exomer, and this drop was placed on a cover slip above the precipitant solution. Hexagonal plate-shaped crystals appeared after 5–7 days. Crystals were cryoprotected in 0.3 M ammonium sulfate, 0.1 M citric acid pH 4.0, 30% glycerol, and 100 μ M peptide. Diffraction data were collected at CHESS (Cornell High Energy Synchrotron Source) beamline A1 and processed using HKL-2000 (Otwinowski and Minor, 1997). The structure was solved by molecular replacement with Phaser in the PHENIX software suite (Adams et al., 2010) using residues 1–77 of Chs5 and all of Chs6 from the Chs5(1–299)/Chs6 exomer complex structure (PDB: 4GNS; Paczkowski et al., 2012). Density for the Chs3 peptide was clearly visible in initial difference maps (**Figure 6A**). The model was refined by manual adjustment in Coot (Emsley et al., 2010) and refinement in PHENIX. Our software is maintained by SBGrid (Morin et al., 2013).

The coordinates and structure factors have been deposited in the Protein Data Bank (www.rcsb.org) with accession number 4U9T.

## Results

### The N-terminus of Chs3 is important for function and localization

**Figure 1:**
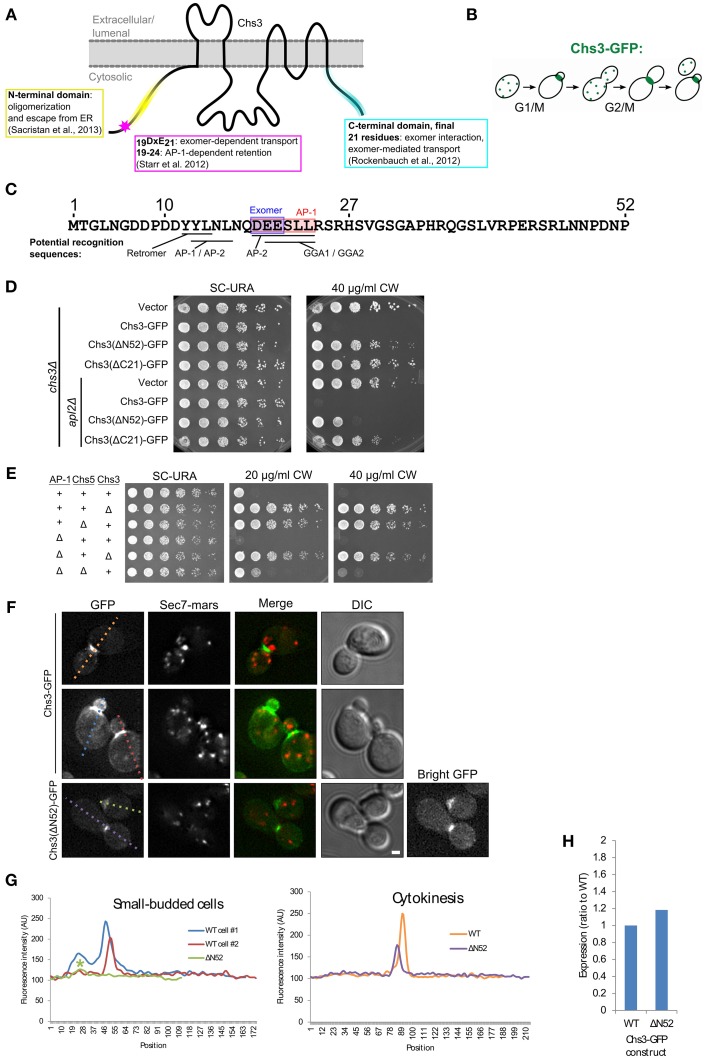
**The N-terminus of Chs3 is important for its function and localization. (A)** The topology of Chs3 (Sacristan et al., 2013) and regions known to be important for its trafficking. **(B)** The itinerary of Chs3 throughout the cell cycle. **(C)** The N-terminus of Chs3 with sites known to be required for adaptor protein interaction (shaded rectangles) and predicted sorting signals for other cargo adaptors. **(D)** Chs3-GFP mutant plasmids were transformed into both a *chs3*Δ *SEC7-Mars* strain and a *chs3*Δ *SEC7-Mars apl2*Δ strain and a 10-fold dilution series was plated on indicated media. Plates were imaged after 2 days at 30°C. See also Figure S2 for additional CW concentrations. **(E)** Strains with indicated phenotypes were plated on indicated concentrations of CW. All strains are genomic *chs3*Δ. Chs3 “+” strains contain Chs3-GFP plasmid, and other strains contain empty expression vector to support growth on SC-URA. See also Figure S2 for additional CW concentrations. **(F)** Chs3-GFP and Sec7-Mars localization in *chs3*Δ cells. Wild type cells showing bud neck localization in small buds (first row) and at cytokinesis (second row) are represented. Chs3(ΔN52)-GFP intensity at bud neck is very low, so a “bright GFP” image with light levels scaled to increase visibility is included. Scale bar, 1 μm. **(G)** Line profile of intensity along dotted lines in (F) of cells with small buds (left) or cytokinesis (right). Asterisk marks significantly lower peak at the bud neck of Chs3(ΔN52)-GFP localization. **(H)** Protein expression level of Δ52 construct relative to wild type Chs3-GFP, as calculated from the α-GFP Western blots in Figure S1, normalized to loading control. Value shown is an average between the two experiments.

Chs3 is a polytopic membrane protein with both termini exposed to the cytoplasm (Figure 1A). The cell cycle dependent localization of Chs3 (Figure 1B) depends upon its transit through multiple trafficking pathways. Several potential sorting signals reside in the N-terminus of Chs3 (Figure 1C).

To assess the importance of the N- and C-termini of Chs3 in its trafficking, we transformed truncated forms of the Chs3 protein into a strain in which Chs3 had been deleted. Chs3 transport defects were assayed by resistance to calcofluor white (CW), which indicates decreased levels of chitin in the cell wall. Increased resistance to CW arises through inactivation or mislocalization of Chs3. All mutations were made in a Chs3-GFP plasmid, which was found to be functional by its ability to rescue normal CW sensitivity levels. Deleting either residues 2–52 (ΔN52) or 1145–1165(ΔC21) of Chs3 caused significant CW resistance (Figure 1D).

Disrupting the AP-1 clathrin adaptor complex impairs Chs3 retention within the cell, and therefore will partially rescue mutants that are compromised specifically in TGN to PM trafficking (Figure 1E) (Valdivia et al., 2002). CW sensitivity of a strain containing Chs3(ΔN52)-GFP was rescued by deletion of the AP-1 component *APL2* (Figure 1D), suggesting this truncation disrupts transport to the PM. In contrast, CW sensitivity of the Chs3(ΔC21)-GFP mutant was not rescued by the AP-1 disruption (Figure 1D). This suggests an additional unknown role for the C-terminus of Chs3 in trafficking or activation, in addition to its identified role interacting with the exomer complex (Rockenbauch et al., 2012). Together, these results indicate that both the N-terminus and C-terminus of Chs3 are important for its trafficking, though they play different roles.

We examined the localization of the N-terminally truncated Chs3-GFP relative to that of the wild type Chs3-GFP. While Chs3 is seen at the bud neck in small buds and at the septum during cytokinesis in both the mutant and wild type strains (Figure 1F), the fluorescence intensity at these structures was lower for the Chs3(ΔN52)-GFP than the wild type (Figures 1F,G). Surprisingly, there are fewer visible internal punctae of GFP containing the truncated protein, suggesting an additional defect in retention at the TGN. To determine if this mutant protein has reduced expression relative to the wild-type, we performed α-GFP Western blot analysis and observed that the expression level of the mutant appears similar to that of the wild-type protein (Figure 1H, Figure S1). Deletion of residues 1-63 of Chs3 was previously reported to result in Chs3 being retained in the ER (Sacristan et al., 2013), but we did not observe any ER-retention of the ΔN52 mutant. It is unclear where in the cell the remaining ΔN52 protein is being retained when not transported to the bud neck, since it is not visibly enriched in any compartments. It is possible that it is not visible because it is spread out over multiple locations, perhaps partially retained in the TGN and endosomes and spread diffusely along the PM. Overall, residues 1–52 appear to play a role in efficient transport to the bud neck, but their deletion does not completely prevent transport to the PM.

### Distinct regions of the Chs3 N-terminus mediate different trafficking pathways

**Figure 2:**
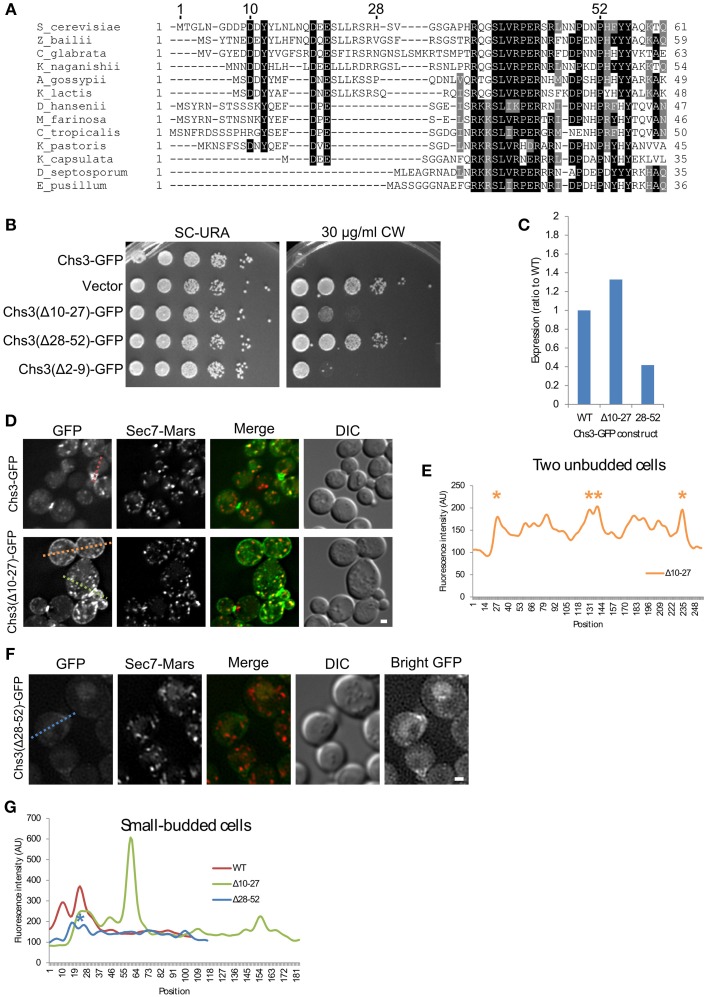
**Residues 10–27 and 28–52 are important for distinct trafficking steps**. **(A)** Multiple sequence alignment of several Chs3 homologs. Residues are shaded to highlight similarity (gray) or identity (black). **(B)** The indicated plasmids were transformed into both a *chs3*Δ*SEC7-Mars* strain and a *chs3*Δ*SEC7-Mars apl2*Δ strain and plated on indicated media. Plates were imaged after 2 days at 30°C. See also Figure S2 for additional CW concentrations. **(C)** Protein expression level of mutated Chs3-GFP constructs relative to wild type as calculated from the α-GFP Western blots in Figure S1, normalized to loading control. **(D)** Chs3-GFP and Sec7-Mars localization in *chs3*Δ cells for WT Chs3-GFP and Chs3(Δ10–27)-GFP, at equivalent light levels. Scale bar, 1 μm. **(E)** Line profile of intensity along dotted lines in **(D)** of unbudded cells. Asterisks mark higher localization of Chs3(Δ10–27)-GFP to the plasma membrane at cell boundaries. **(F)** Chs3(Δ28–52)-GFP localization examined as in **(C)**. Additional “Bright GFP” image with light levels scaled to improve visibility is presented. **(G)** Line profile as in **(E)** for small-budded cells. Asterisk indicates lower bud neck localization of Chs3(Δ28–52)-GFP.

Since the deletion of residues 2–52 may remove multiple sorting signals (Figure 1C), shorter segments within residues 1–52 (Figure 2A) were deleted to more precisely map the function of the N-terminus. Deletions comprising residues 2–9, 10–27, or 28–52 all conferred some resistance to CW (Figure 2B). Deletion of residues 2-9 had the mildest phenotype. Deletion of residues 10–27, which contain residues reported to interact with exomer and AP-1 (Starr et al., 2012), had a moderate phenotype. The deletion of residues 28–52 had the greatest effect, nearly equivalent to that of a strain lacking Chs3. This Chs3(Δ28–52)-GFP construct also had somewhat lower expression than the wild type or Chs3(Δ10–27)-GFP, which may contribute to the strong CW growth phenotype (Figure 2C, Figure S1). These results suggest that residues 28–52 are the most important in this region for the transport of Chs3 to the PM.

The Chs3(Δ10–27)-GFP protein localized to small buds and the primary septum as well as the wild type (Figure 2D). However, it also mislocalized to large portions of the PM, especially in non-budded and large-budded cells (Figures 2D,E). This suggests a defect in internalization by endocytosis. Chs3 enzymatic activity requires activation by Chs4, which is held at the bud neck by an indirect interaction with septins (DeMarini, 1997). Therefore, the mutant Chs3 mislocalized throughout the PM is unlikely to be actively producing chitin, likely causing the increased CW resistance we observed.

The Chs3(Δ28–52)-GFP mutant exhibited a different pattern of localization (Figure 2F). It localized to the bud neck and septum, but with lower fluorescence intensity than the wild type protein (Figure 2G). We also observed GFP in the vacuole with this mutant, and less localization to TGN punctae. The inability to divert endocytosed Chs3(Δ28–52)-GFP back to the TGN and away from the vacuole could explain the lower level of this protein in cells (Figure 2C, Figure S1).

### Mutation of residues 19–21 leads to increased PM localization

**Figure 3:**
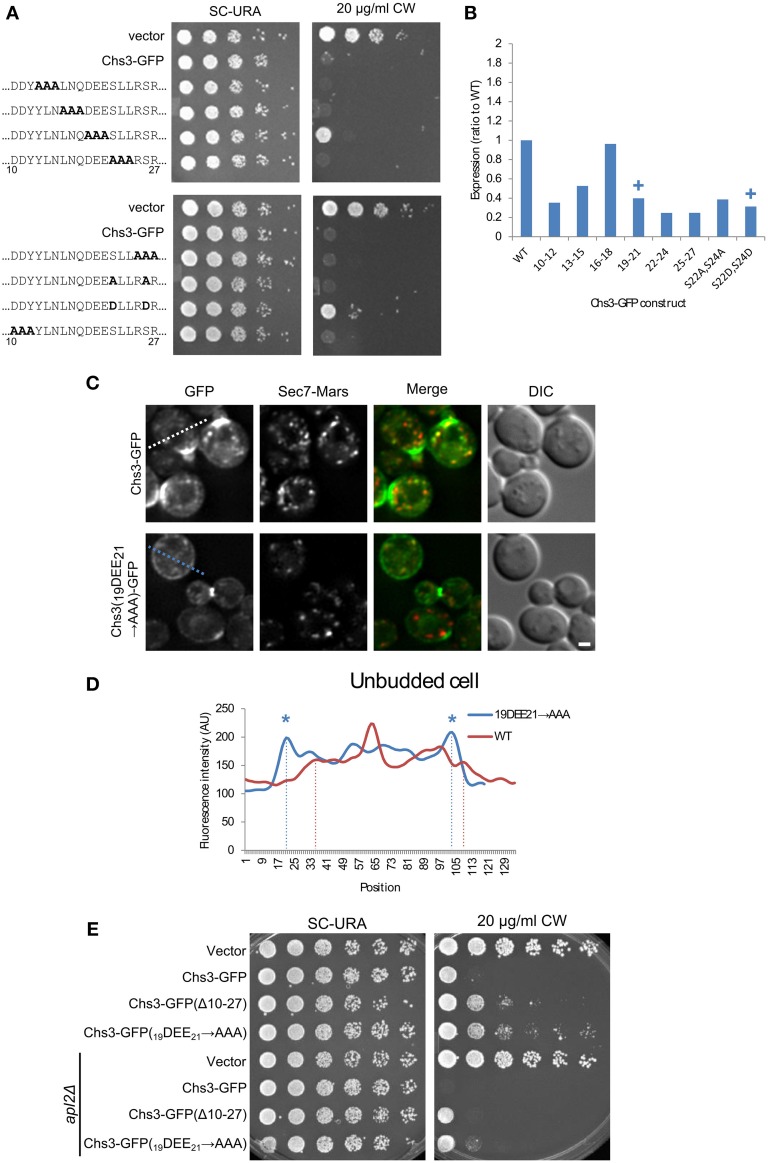
**Mutation of Chs3 residues 19–21 causes a defect in internalization**. **(A)** Chs3-GFP plasmids containing alanine scanning mutants were transformed into a *chs3*Δ*SEC7-Mars* strain and plated on indicated concentrations of CW. Plates were imaged after 2 days at 30°C. See also Figure S2 for additional CW concentrations. **(B)** Protein expression level of alanine-scanning Chs3-GFP constructs relative to wild type as calculated from the α-GFP Western blots in Figure S1, normalized to loading control. Mutations that confer CW resistance phenotypes are marked with (+). **(C)** GFP and Sec7-Mars localization in *chs3*Δ cells for WT Chs3-GFP and Chs3(_19_DEE_21_-**→** AAA)-GFP, at equivalent light levels. Scale bar, 1 μm. **(D)** Line profile of intensity along dotted line in **(C)** of unbudded cell expressing mutant protein, or across non-bud section of cell expressing WT protein. Asterisks mark higher localization of Chs3(_19_DEE_21_ **→** AAA)-GFP to the plasma membrane at cell boundaries (dotted lines). **(E)** Chs3(Δ10–27)-GFP and Chs3(_19_DEE_21_ **→** AAA)-GFP were introduced into the *chs3*Δ and *chs3*Δ*apl2*Δ strains and plated on indicated media. Plates were imaged after 2 days at 30°C. See also Figure S2 for additional CW concentrations.

To identify the residues within the 10–27 segment most important for its function, alanine scanning mutagenesis was performed, with groups of three sequential amino acids mutated to alanine. In addition, the two serine residues were mutated, as serine is a potential phosphorylation target. The serines were mutated to either alanine or aspartate, to prevent or mimic phosphorylation, respectively. Two of these mutants had a mild CW resistance phenotype: _19_DEE_21_ **→** AAA, and S24D/S26D (Figure 3A). While the mutated constructs varied in expression level, the mutant constructs with the lowest levels were still sensitive to CW, indicating all mutants were expressed at levels sufficient to maintain normal levels of chitin in the cell wall if trafficked properly (Figure 3B, Figure S1).

Since a role for residues 19–21 in exomer-mediated transport is consistent with previous findings (Starr et al., 2012), we also looked at the localization of this mutant protein (Figures 3C,D). Its pattern of localization appeared the same as that of the deletion of residues 10–27 (Figure 2D): normal localization at the bud neck, and mislocalization along the entire PM. This suggests that residues 19–21 are important for internalization and possibly retention of Chs3. Previously these residues were shown to be important for the interaction between Chs3 and AP-1 (Starr et al., 2012), and it is possible they are also important for the interaction of Chs3 with AP-2 during endocytosis, as AP-1 and AP-2 recognize similar sorting signals (Bonifacino and Traub, 2003).

We next determined whether disruption of AP-1 function would rescue the CW sensitivity of the Chs3(Δ10–27)-GFP or Chs3(_19_DEE_21_ **→** AAA)-GFP mutants. AP-1 disruption did increase the CW sensitivity of both mutants (Figure 3E). This result indicates that AP-1 contributes to retention of the Chs3(_19_DEE_21_ **→** AAA)-GFP mutant, and implies AP-1 can still partially interact with this mutant despite disruption of this motif.

### Mutation of residues 41–43 leads to decreased PM localization

**Figure 4:**
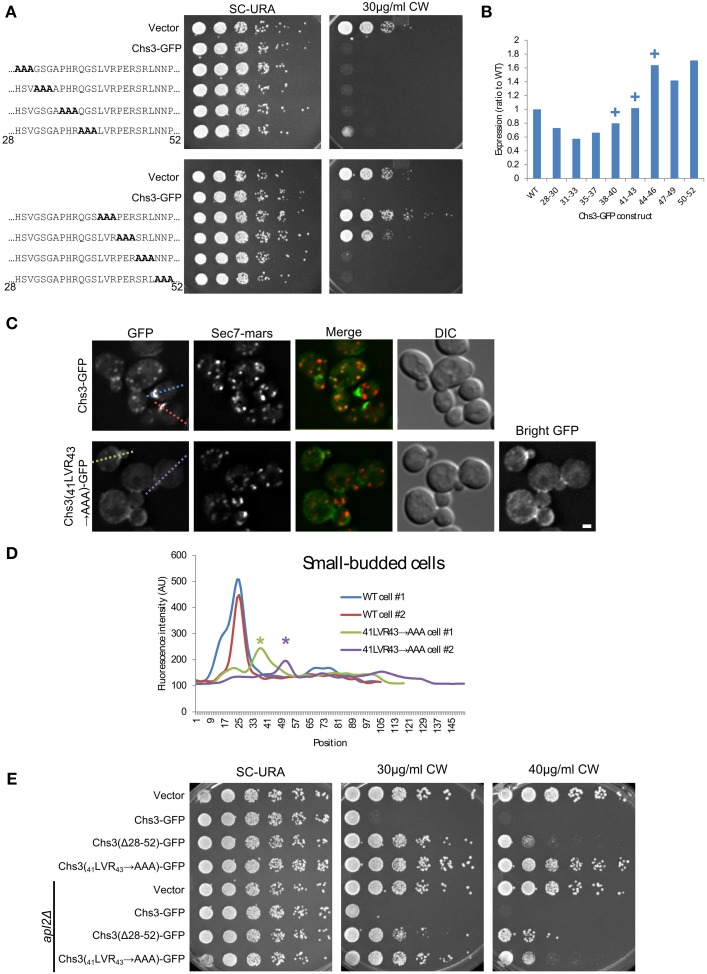
**Deletion of Chs3 residues 41–43 has a strong defect in transport**. **(A)** Chs3-GFP plasmids containing alanine scanning mutants were transformed into a *chs3ΔSEC7-Mars* strain and plated on indicated concentrations of CW. Plates were imaged after 2 days at 30°C. See also Figure S2 for additional CW concentrations. **(B)** Protein expression level of alanine-scanning Chs3-GFP constructs relative to wild type as calculated from the α-GFP Western blots in Figure S1, normalized to loading control. Mutations that confer CW resistance phenotypes are marked with (+). **(C)** GFP and Sec7-Mars localization in *chs3*Δ cells for WT Chs3-GFP and Chs3(_41_LVR_43_ **→** AAA)-GFP, at equivalent light levels. Scale bar, 1 μm. **(D)** Line profile of intensity along dotted lines in **(C)** of small-budded cells. Asterisks mark significantly lower peaks at the bud neck of Chs3(_41_LVR_43_ **→** AAA)-GFP localization. **(E)** Chs3(Δ28–52)-GFP and Chs3(_41_LVR_43_ **→** AAA)-GFP were introduced into the *chs3*Δ and *chs3*Δ*apl2*Δ strains and plated on indicated media. Plates were imaged after 2 days at 30°C. See also Figure S2 for additional CW concentrations.

We used alanine scanning mutagenesis to determine the most important portions of the segment containing Chs3 residues 28–52. The mutation of residues 41–43 resulted in the strongest CW resistance, equivalent to that of the ΔN52 truncation (Figure 4A). Mutations in residues immediately preceding or following these residues (38–40 and 44–46) resulted in more modest CW resistance phenotypes. This indicates a region centered around residues 41–43 is required for normal transport or function of Chs3. Importantly, this region is highly conserved (Figure 2A). The defect of this mutant is not due to lower levels of the protein, as the _41_LVR_43_ **→** AAA mutation did not decrease expression (Figure 4B, Figure S1). The _41_LVR_43_ **→** AAA mutation affects Chs3 localization, with decreased levels at the bud neck and the septum (Figures 4C,D), with no apparent increase in retention at the TGN. This localization defect was slightly less severe than deleting residues 28–52. This could indicate the residues around 41–43 are still able to facilitate transport even when 41–43 are mutated, perhaps by interacting with exomer.

Surprisingly, the Chs3(_41_LVR_43_ **→** AAA)-GFP mutant was more resistant to CW than the Chs3(Δ28–52)-GFP mutant (Figure 4E). This may suggest a more drastic local structural rearrangement when these residues are mutated to alanine vs. what occurs when the entire segment is deleted. Alternatively, more than one trafficking pathway may be disrupted by deletion of the entire segment. The Chs3(_41_LVR_43_ **→** AAA)-GFP mutant was partially rescued by the *apl2* deletion (Figure 4E), indicating it is still able to interact with AP-1 and suggesting a role for this region in transport to the PM. In contrast, the CW sensitivity of the Chs3(Δ28–52)-GFP mutant was not rescued by *apl2* deletion. This suggests this mutant is not efficiently retained by AP-1, and is consistent with our observation that this mutant is degraded in the vacuole (Figure 2E).

### Residues 28–52 interact with exomer more strongly than residues 10–27 or the C-terminus

**Figure 5:**
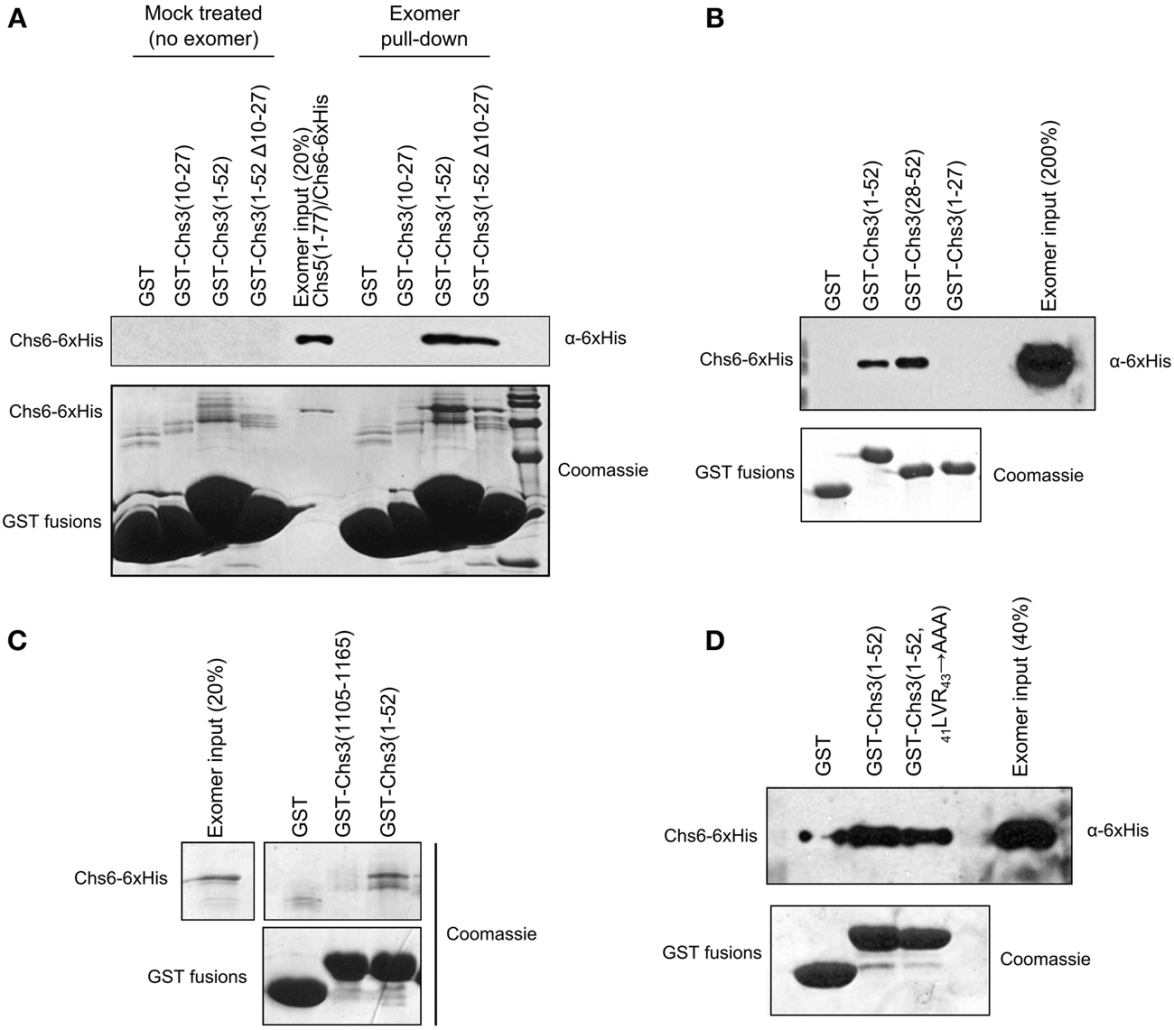
**Residues 28–52 interact with exomer *in vitro***. **(A)** *In vitro* interaction assay to measure ability of GST-tagged Chs3 fragments to interact with purified exomer complex. Exomer was detected by α-6xHis antibody which recognized His-tagged Chs6. GST-tagged constructs were detected by Coomassie. In the “mock treated” samples, the final exomer purification buffer was added in place of exomer. **(B)** Chs3 fragment pull-down assay as described in **(A)** comparing the two halves of the Chs3(1–52) segment. **(C)** Assay as described in **(A)** to compare interaction of N- and C-termini, but with bound exomer detected by Coomassie staining. **(D)** Assay as described in **(A)** to analyze effect of _41_LVR_43_ **→** AAA mutation on interaction.

We created an *in vitro* assay to detect the direct interaction of Chs3 fragments with exomer. GST-fusion proteins were immobilized on glutathione resin, which was incubated with exomer complex. Residues 1–52 were able to interact with exomer (Figure 5A). Residues 10–27 did not interact with exomer in this assay, and deleting residues 10–27 from the residue 1–52 segment only decreased exomer binding by about half. Together, these indicate residues 10–27 are neither necessary nor sufficient for interaction in this assay. Residues 28–52 were able to interact with exomer even more strongly than residues 1–52 (Figure 5B).

A segment at the C-terminus of Chs3 was unable to interact with exomer in this assay (Figure 5C), despite previously published evidence that it does interact with exomer (Rockenbauch et al., 2012). The previous report detected an interaction with exomer from cell lysates, rather than with purified exomer as used in our assay, so it is possible that the interaction requires another binding partner or a post-translational modification to occur. Alternatively, a more complete Chs5 protein may be required for the interaction, since the exomer complex used here contained only residues 1–77 of Chs5. This truncated Chs5 is not fully functional, but it contains all the structural components necessary to homodimerize and interact with ChAP proteins (Paczkowski et al., 2012), which should allow the exomer complex to interact normally with cargo *in vitro*. Alternatively, post-translational modification of exomer or Chs3 may regulate the interaction.

Since the _19_DEE_21_ **→** AAA mutation had such a strong effect on Chs3 trafficking, we tested its effect on the *in vitro* interaction between Chs3(1–52) and purified exomer. Surprisingly, this mutated fragment interacted as well as the wild-type GST-Chs3(1–52) fragment (Figure 5D). The assay may not be sensitive enough to observe a slight reduction in binding affinity, which could strongly affect Chs3 trafficking *in vivo*.

### Residues 10–27 interact with exomer based on structural analysis

**Figure 6:**
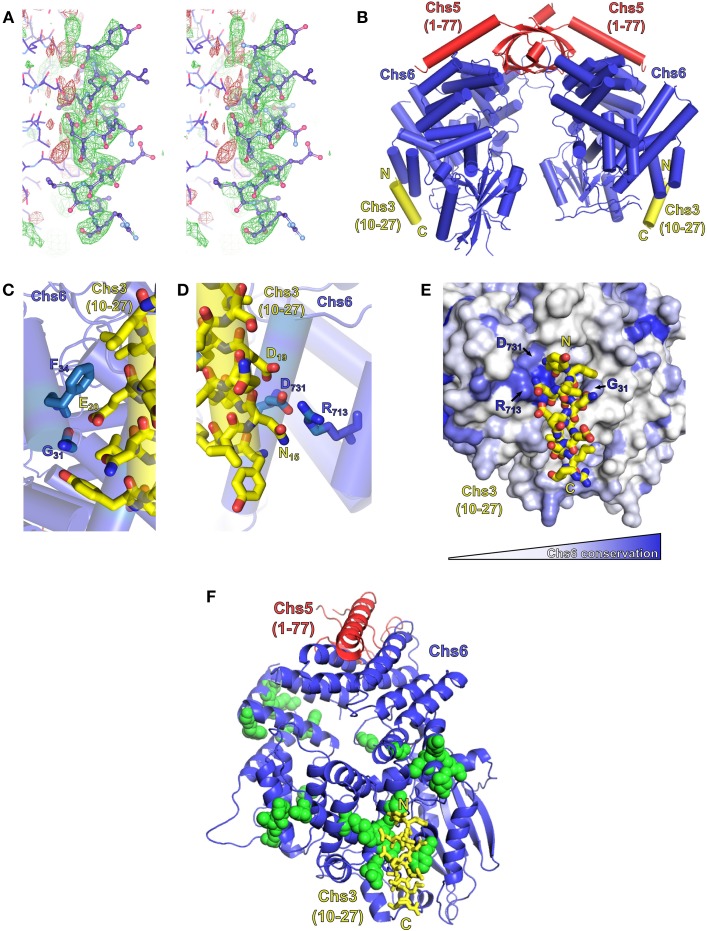
**Exomer co-crystalizes with Chs3 residues 10–27**. **(A)** Stereo image (wall-eyed) of the original F_o_-F_c_ difference map calculated after molecular replacement, used to model the Chs3 peptide (shown as ball-and-stick model), contoured at +2.5 σ (green) and −2.5 σ (red). The negative density to the left of the peptide is assumed to be due to series truncation errors arising from the modest resolution of the data. **(B)** Model of an exomer complex containing Chs5(1–77) (red), Chs6-6xHis (blue) and a peptide of Chs3 residues 10–27 (yellow, only residues 12–25 are visible). Model was created using Chs5(1–77)/Bch1 structure (PDB ID: 4IN3) (Richardson and Fromme, 2013) as a template, replacing each Bch1 subunit with the structure of Chs6 and peptide from this study. **(C,D)** Close-up views of the interaction of the Chs3 peptide with Chs6. **(E)** Chs6 colored by conservation within Chs6 and Bch2 ChAP proteins, white to blue representing low to high conservation. **(F)** Structure rotated 90° from **(A)**, showing location of Chs6 mutations that had no effect on Chs3 trafficking (green spheres), as listed in Table 2. This panel also depicts the crystallographic asymmetric unit.

Obtaining a crystal structure of exomer bound to its sorting signal within Chs3 would elucidate both the location of binding within Chs6, and the structure of that segment of Chs3. Therefore, we attempted to co-crystallize exomer with several peptides from the N- and C-termini of Chs3. Most crystals diffracted well but did not contain the peptide. After screening many crystals, several were found that contained the peptide matching residues 10–27. This peptide formed an alpha-helical structure, and bound to a region on the surface of Chs6 roughly opposite where Chs5 binds (Figures 6A,B; Table 1). This observed helix is 14 residues in length, and we found residues 12–25 to be the best fit to the density (Figure S3). This indicates residues 10–27 can interact directly with exomer at high concentrations, despite the lack of binding seen in our *in vitro* assay (Figure 5B). It is possible other peptides that interact more strongly with exomer disrupted crystal contacts or destabilized the structure, leading to their exclusion from the crystals. This is supported by our observation that when a peptide containing residues 1–52 of Chs3 was added to the cryoprotectant solution, the exomer crystals dissolved. We also note that no additional electron density was visible when crystals were soaked with a peptide corresponding to residues 1128–1165 of the Chs3 C-terminus.

**Table 1:** **Crystallographic statistics**.

Wavelength (Å)	0.9767
Resolution range (Å)	44.65−2.59 (2.683−2.59)
Space group	P 63 2 2
Unit cell	a = 218.613 Å, b = 218.613 Å, c = 137.848 Å α = 90°, β = 90°, γ = 120°
Total reflections	59,962
Unique reflections	59936 (5730)
Multiplicity	12.2 (10.0)
Completeness (%)	99.00 (96.33)
<I>/< σ (I) >	19.45 (5.06)
Wilson B-factor	50.18
R_sym_	0.090 (0.717)
R_cryst_	0.1796 (0.2202)
R_free_	0.2107 (0.2534)
Number of atoms	6100
Protein (755 residues)	6008
Water	92
RMS (bonds) (Å)	0.006
RMS (angles) (°)	1.09
Ramachandran favored (%)	94
Ramachandran outliers (%)	1.5
Clash score	7.88
Average B-factor	68.20
Protein	68.40
Water	55.60

Values in parentheses represent the highest resolution shell.

The Chs3 peptide makes direct contact with several residues on the surface of Chs6 (Figures 6C–E). Chs3 residue D19 appears to participate in a hydrogen bonding network with Chs6 residues D731 and R713. Chs3 residue E20 appears to hydrogen bond with the main chain carbonyl oxygen of Chs6 residue G31. Both of these Chs3 residues, D19 and E20, are part of the conserved DEESLL sequence that was previously found to be important for interaction with AP-1 (Starr et al., 2012) and that we found to be important for endocytosis of Chs3 (Figure 3C).

Based on the crystal structure, several mutations were made in the Chs6 protein in an attempt to disrupt the interaction (Figure 6F, Table 2). These mutated proteins, in addition to many mutations made based on sequence conservation (Figure 6E), were still able to mediate transport of Chs3. This supports our hypothesis that residues 10–27 are not required for transport of Chs3 to the PM, but rather play a more important role in endocytosis of Chs3. Nevertheless, the structure reveals how this clathrin adaptor motif can be bound by exomer, perhaps providing a basis for competition between adaptors at the TGN.

**Table 2:** **Many Chs6 mutations have no phenotype**.

**Plasmid name**	**Residues mutated**
**MUTATIONS MADE BEFORE CRYSTAL STRUCTURE**	
pAS62	Wild-type Chs6-Myc
pJC1	G540A W541A
pJC2	R548E F552A
pJC3	C582A W585A D587K
pAS70	S237A
pAS71	S253A
pAS72	T516A
pAS73	S612A
pAS92	C216A K217D K218D
**MUTATIONS BASED ON CRYSTAL STRUCTURE**	
pAS109	R713A
pAS110	D724A
pAS111	V728D
pAS112	D731A
pAS115	R713A V728D
pAS116	R713A D731A
pAS117	D724A D731A
pAS126	V728W
pAS127	A735W
pAS128	V728R
pAS129	A735R
pAS130	V728E
pAS131	A735E
pAS152	V728R A735R
pAS153	V728E A735E
pAS180	K210A
pAS183	R637A T638A
pAS184	D672A
pAS191	F34A
pAS192	F34A R713A
pAS193	F34A D731A

The indicated residues were mutated in a Chs6-Myc plasmid and transformed into a chs6Δstrain. The cells showed no CW resistance. Mutations in italics were made based on the crystal structure in (6A).

## Discussion

We have shown the importance of the N-terminal 52 residues of Chs3 for its transport, as deletion of these residues decreases the amount of Chs3 at the bud neck as well as TGN punctae, and the mutant cells are CW resistant. We also endeavored to more precisely identify sorting signals within this region that interact with exomer and potentially other cargo adaptor proteins. Residues 10–27 contribute to internalization, while residues 28–52 contribute to retrieval of Chs3 from the endosomal system and to exomer-mediated transport to the PM, and are sufficient to interact with exomer *in vitro*. While residues 10–27 did not interact in our *in vitro* pull-down assay, they did interact with exomer in the crystals, suggesting they can contribute to the interaction, though perhaps with low affinity.

**Figure 7:**
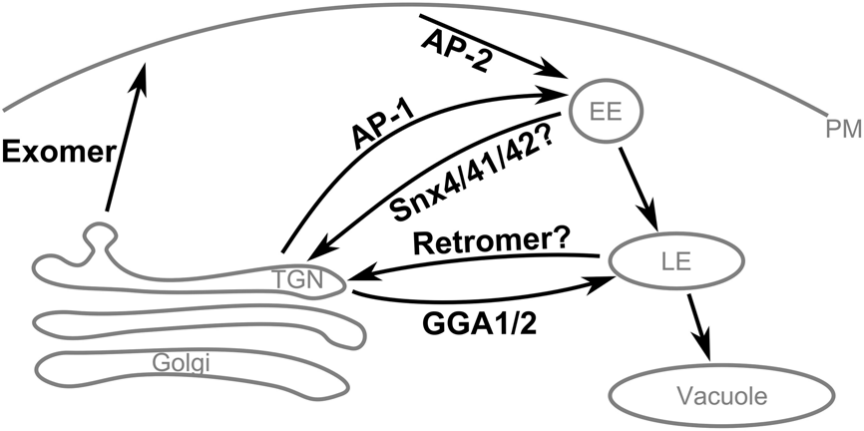
**Summary of Chs3 trafficking pathways**. Chs3 is transported through several intracellular trafficking pathways throughout the cell cycle. As the yeast TGN is not a stable compartment, retention of cargo at the TGN appears to require traffic to endosomes by the AP-1 and Gga1/2 clathrin adaptors and subsequent retrieval back to the TGN, likely via the retromer and/or SNX4/41/42 pathways.

The itinerary of Chs3 involves transport through several pathways in the cell (Table 3, Figure 7). The first step after synthesis of Chs3 is exit from the ER, which requires Chs7 (Trilla, 1999). This process requires the N-terminus of Chs3, since truncation of the first 126 residues prevents ER exit (Sacristan et al., 2013), but Chs3(ΔN52)-GFP exits the ER normally indicating the required residues are located between 52 and 126. After exit from the ER and transit through the Golgi, Chs3 can be transported to the PM by two pathways. The first is exomer-mediated, and requires both the N- and C-termini of Chs3. Our results indicated residues 28–52 are particularly important for this step. The second pathway is not exomer-mediated, and can allow normal levels of Chs3 to reach the PM when retention is disrupted. It is unknown whether this is mediated by any cargo adaptors or requires sorting signals, or whether it is a non-specific inclusion of Chs3 into secretory vesicles. Chs3 endocytosis is mediated by the AP-2 clathrin adaptor. The mislocalization of Chs3(Δ10–27)-GFP and Chs3(_19_DEE_21_ **→** AAA)-GFP to the PM suggests AP-2 may bind the DEESLL motif at residues 19–24. Therefore, AP-1, AP-2, and exomer likely bind to this motif during different stages of Chs3 trafficking. Interestingly, the conformation of this motif when bound to Chs6 is alpha-helical, in contrast to the conformation (D/E)XXXLL motifs adopt when bound to AP-1 and AP-2 (Kelly et al., 2008; Jackson et al., 2010). In those interactions, the (D/E)XXXLL motif is in an extended conformation.

**Table 3:** **Summary of Chs3 mutations analyzed in this study and their effect on trafficking**.

**Mutant**	**CW growth phenotype**	**CW sensitivity rescued by *apl2*Δ**	**Localization phenotype**	**Likely pathways disrupted**
*chs3*Δ	++++	No	N/A	N/A
ΔN52	++++	Strongly	Weak bud neck, few punctae	TGN**→**PM (exomer) TGN**→**Endosomes (AP-1,Gga1/2) Endosomes**→**TGN (Retromer, Snx4/41/42)
_19_DEE_21_**→**AAA	+	Moderately	Normal bud neck and punctae, additional at PM	Endocytosis (AP-2) TGN**→**Endosomes (AP-1,Gga1/2)
Δ10-27	++	Moderately	Normal bud neck and punctae, additional at PM	Endocytosis (AP-2) TGN**→**Endosomes (AP-1,Gga1/2)
Δ28-52	+++	No	Weak bud neck, few punctae, vacuole	TGN**→**PM (exomer) Endosomes**→**TGN (Retromer, Snx4/41/42)
_41_LVR_43_**→**AAA	++++	Moderately	Weak bud neck, few punctae	TGN**→**PM (exomer)
ΔC21	++++	No	TGN punctae only (Rockenbauch et al., 2012)	TGN**→**PM (exomer)

The TGN in yeast is a transient compartment that acts to sort proteins before distributing them to other locations within the cell. Therefore, retention of a protein at the TGN is not a static storage process, but a dynamic process of tubular and vesicular transport from the TGN to the endosomal system and then back to the TGN. Chs3 requires AP-1 and Gga1/2 to transport it into the endosomal system. AP-1 binds Chs3 residues 19–24, but also must interact elsewhere in Chs3, since AP-1 deletion can rescue the CW sensitivity even when residues 1–52 are deleted. Two complexes that mediate transport from the endosomal system to the TGN are the retromer complex and the Snx4/41/42 complex. One or both of these complexes could be required for the retrieval of Chs3 back to the TGN. One of the complexes involved in this step may bind to residues 28–52 of Chs3, as we saw mutations in this region resulted in mislocalization to the vacuole. The lack of vacuolar localization in either larger (Δ52) or smaller (_41_LVR_43_ **→** AAA) disruptions suggests that a very specific set of interactions must be blocked, and others allowed, to result in this phenotype.

Our results suggest a competition of cargo adaptors for overlapping or closely adjacent sorting signals could be used to regulate and balance the pathways required for Chs3 transport. This form of regulation is unlikely to be specific to Chs3, since another exomer cargo, Pin2, contains a potential AP-1 sorting signal within the segment required for exomer-mediated transport (Ritz et al., 2014). An analogous mechanism has been demonstrated for the binding of the myosin V motor to different cargos, although the situation is reversed, in which multiple cargos compete for binding the same site on the motor (Eves et al., 2012).

This complex set of overlapping sorting signals and redundant transport pathways could allow for careful control of Chs3 localization throughout the cell cycle and efficient changes in response to signals. It is possible there are proteins in many organisms that use competitive binding of cargo adaptors to cycle between the cell surface and internal compartments.

### Conflict of interest statement

The authors declare that the research was conducted in the absence of any commercial or financial relationships that could be construed as a potential conflict of interest.
