# Supplementary material for: Distinct N-terminal regions of the exomer secretory vesicle cargo Chs3 regulate its trafficking itinerary
Source: Front Cell Dev Biol. 2014 Sep 3;2:47. doi: 10.3389/fcell.2014.00047 (PMC4207043; doi:10.3389/fcell.2014.00047)
Supplement: Supplementary file 1 [file DataSheet1.DOCX]

***Supplementary Material***

**Distinct N-terminal regions of the exomer secretory vesicle cargo Chs3 regulate its trafficking itinerary**

**Amanda M. Weiskoff^1^, J. Christopher Fromme^1^***

^1^Weill Institute for Cell and Molecular Biology, Department of Molecular Biology and Genetics, Cornell University, Ithaca, NY, USA

*** Correspondence:**

J. Christopher Fromme

Weill Institute for Cell and Molecular Biology

Department of Molecular Biology and Genetics

Cornell University

457 Weill Hall

Ithaca, NY, 14853, USA

jcf14@cornell.edu

1. **Supplementary Figures and Tables**

## Suplementary Tables

**Supplementary Table 1. Yeast strains used in this study.**

| **Name** | **Genotype** | **Source** |
| --- | --- | --- |
| SEY6210 | MATα his3-Δ200 leu2-3,112 lys2-801 trp1-Δ901 ura3-52 suc2-Δ9 | (Robinson and Klionsky, 1988) |
| SEY6210.1 | MATa his3-Δ200 leu2-3,112 lys2-801 trp1-Δ901 ura3-52 suc2-Δ9 | (Robinson and Klionsky, 1988) |
| CFY1328 | SEY6210 Sec7-Mars::TRP1 chs3Δ::NatMX | This study |
| CFY1331 | SEY6210 Sec7-Mars::TRP1 chs3Δ::NatMX apl2∆::KanMX | This study |
| CFY209 | SEY6210.1 chs3∆::KanMX | This study |
| CFY247 | SEY6210.1 chs5∆::KanMX | (Paczkowski et al., 2012) |
| CFY267 | SEY6210.1 apl2∆::KANMX | This study |
| CFY1863 | SEY6210 Sec7-Mars::TRP1 chs3Δ::NatMX chs5Δ::His3 | This study |
| CFY1864 | SEY6210 Sec7-Mars::TRP1 chs3Δ::NatMX apl2∆::KANMX chs5Δ::His3 | This study |

## Supplementary Table 2. Plasmids used in this study.

| **Name** | **Description/Protein expressed** | **Vector Backbone** | **Source** |
| --- | --- | --- | --- |
| pRS416 | Centromeric URA3 plasmid |  | (Sikorski and Hieter, 1989) |
| pRB259 | Chs3-GFP | pRS416 | Schekman Lab (unpublished) |
| pAS114 | Chs3(∆10-27)-GFP | pRS416 | This study |
| pAS118 | Chs3(13-15→AAA)-GFP | pRS416 | This study |
| pAS119 | Chs3(16-18→AAA)-GFP | pRS416 | This study |
| pAS120 | Chs3(19-21→AAA)-GFP | pRS416 | This study |
| pAS121 | Chs3(22-24→AAA)-GFP | pRS416 | This study |
| pAS122 | Chs3(25-27→AAA)-GFP | pRS416 | This study |
| pAS123 | Chs3(22,26 S→A)-GFP | pRS416 | This study |
| pAS124 | Chs3(22,26 S→D)-GFP | pRS416 | This study |
| pAS125 | Chs3(10-12→AAA)-GFP | pRS416 | This study |
| pAS140 | Chs3(W1145*)-GFP | pRS416 | This study |
| pAS163 | Chs3-GFP(∆2-52)-GFP | pRS416 | This study |
| pAS165 | Chs3(28-30→AAA)-GFP | pRS416 | This study |
| pAS166 | Chs3(31-33→AAA)-GFP | pRS416 | This study |
| pAS167 | Chs3(35-37→AAA)-GFP | pRS416 | This study |
| pAS168 | Chs3(38-40→AAA)-GFP | pRS416 | This study |
| pAS169 | Chs3(41-43→AAA)-GFP | pRS416 | This study |
| pAS170 | Chs3(44-46→AAA)-GFP | pRS416 | This study |
| pAS171 | Chs3(47-49→AAA)-GFP | pRS416 | This study |
| pAS172 | Chs3(50-52→AAA)-GFP | pRS416 | This study |
| pAS186 | Chs3(∆28-52)-GFP | pRS416 | This study |
| pAS188 | Chs3(∆2-9)-GFP | pRS416 | This study |
| pAS62 | Chs6-Myc | pRS416 | This study |
| pJC1 | Chs6(G540A W541A)-Myc | pRS416 | This study |
| pJC2 | Chs6(R548E F552A)-Myc | pRS416 | This study |
| pJC3 | Chs6(C582A W585A D587K)-Myc | pRS416 | This study |
| pAS70 | Chs6(S237A)-Myc | pRS416 | This study |
| pAS71 | Chs6(S253A)-Myc | pRS416 | This study |
| pAS72 | Chs6(T516A)-Myc | pRS416 | This study |
| pAS73 | Chs6(S612A)-Myc | pRS416 | This study |
| pAS92 | Chs6(C216A K217D K218D)-Myc | pRS416 | This study |
| pAS109 | Chs6(R713A)-Myc | pRS416 | This study |
| pAS110 | Chs6(D724A)-Myc | pRS416 | This study |
| pAS111 | Chs6(V728D)-Myc | pRS416 | This study |
| pAS112 | Chs6(D731A)-Myc | pRS416 | This study |
| pAS115 | Chs6(R713A V728D)-Myc | pRS416 | This study |
| pAS116 | Chs6(R713A D731A)-Myc | pRS416 | This study |
| pAS117 | Chs6(D724A D731A)-Myc | pRS416 | This study |
| pAS126 | Chs6(V728W)-Myc | pRS416 | This study |
| pAS127 | Chs6(A735W)-Myc | pRS416 | This study |
| pAS128 | Chs6(V728R)-Myc | pRS416 | This study |
| pAS129 | Chs6(A735R)-Myc | pRS416 | This study |
| pAS130 | Chs6(V728E)-Myc | pRS416 | This study |
| pAS131 | Chs6(A735E)-Myc | pRS416 | This study |
| pAS152 | Chs6(V728R A735R)-Myc | pRS416 | This study |
| pAS153 | Chs6(V728E A735E)-Myc | pRS416 | This study |
| pAS180 | Chs6(K210A)-Myc | pRS416 | This study |
| pAS183 | Chs6(R637A T638A)-Myc | pRS416 | This study |
| pAS184 | Chs6(D672A)-Myc | pRS416 | This study |
| pAS191 | Chs6(F34A)-Myc | pRS416 | This study |
| pAS192 | Chs6(F34A R713A)-Myc | pRS416 | This study |
| pAS193 | Chs6(F34A D731A)-Myc | pRS416 | This study |
| pGEX-2T | GST gene fusion expression vector | pRS416 | GE Healthcare |
| pAS137 | GST-Chs3(10-27) | pGEX-2T | This study |
| pAS138 | GST-Chs3(1105-1165) | pGEX-2T | This study |
| pAS151 | GST-Chs3(1-52) | pGEX-2T | This study |
| pAS154 | GST-Chs3(1-52, ∆10-27) | pGEX-2T | This study |
| pAS176 | GST-Chs3(1-52, 41-43→AAA) | pGEX-2T | This study |
| pAS187 | GST-Chs3(1-27) | pGEX-2T | This study |
| pAS189 | GST-Chs3(1-52, ∆2-9) | pGEX-2T | This study |
| pAS190 | GST-Chs3(28-52) | pGEX-2T | This study |
| pETDuet-1 | T7-promoter driven expression plasmid |  | Novagen |
| pBCR402 | Chs5(1-77)/Chs6-6xHis | pETDuet-1 | (Richardson and Fromme, 2013) |

##
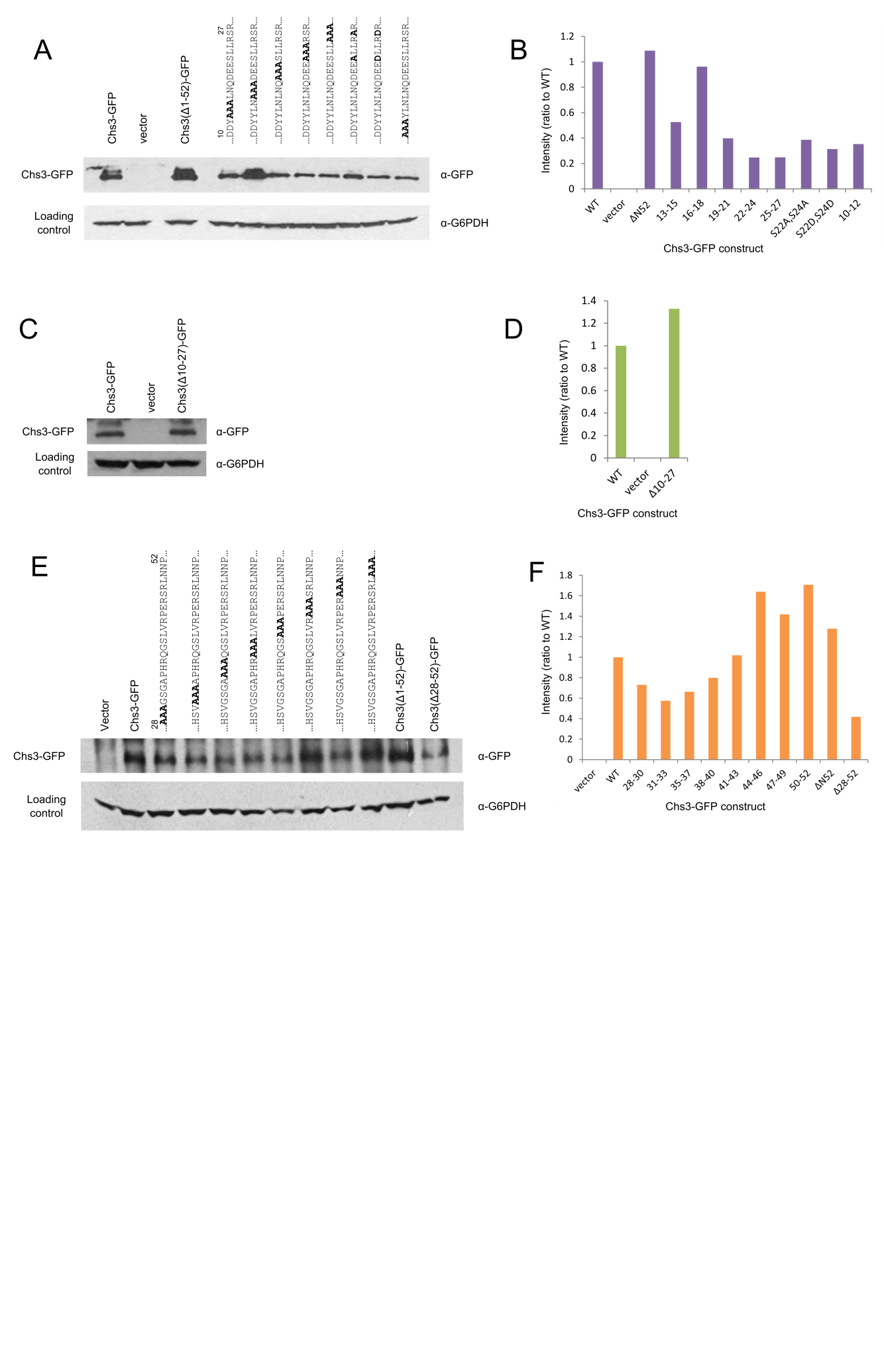
Supplementary Figures

Supplementary Figure 1. Expression of Chs3-GFP constructs. (A) Expression levels of mutated proteins from alanine-scanning in residue 10-27 region compared by α-GFP Western blot. (B) Expression levels shown in (A) quantified by measuring band intensity, subtracting vector-only lane, normalizing by loading control, and calculating ratio of indicated mutant to WT. (C) Expression level of Δ10-27 construct was assayed as in (A). (D) Quantification of (C) as described. (E) Expression levels of mutated proteins from alanine-scanning in residue 28-52 region were compared by α-GFP Western blot. (F) Quantification of (E) as described.


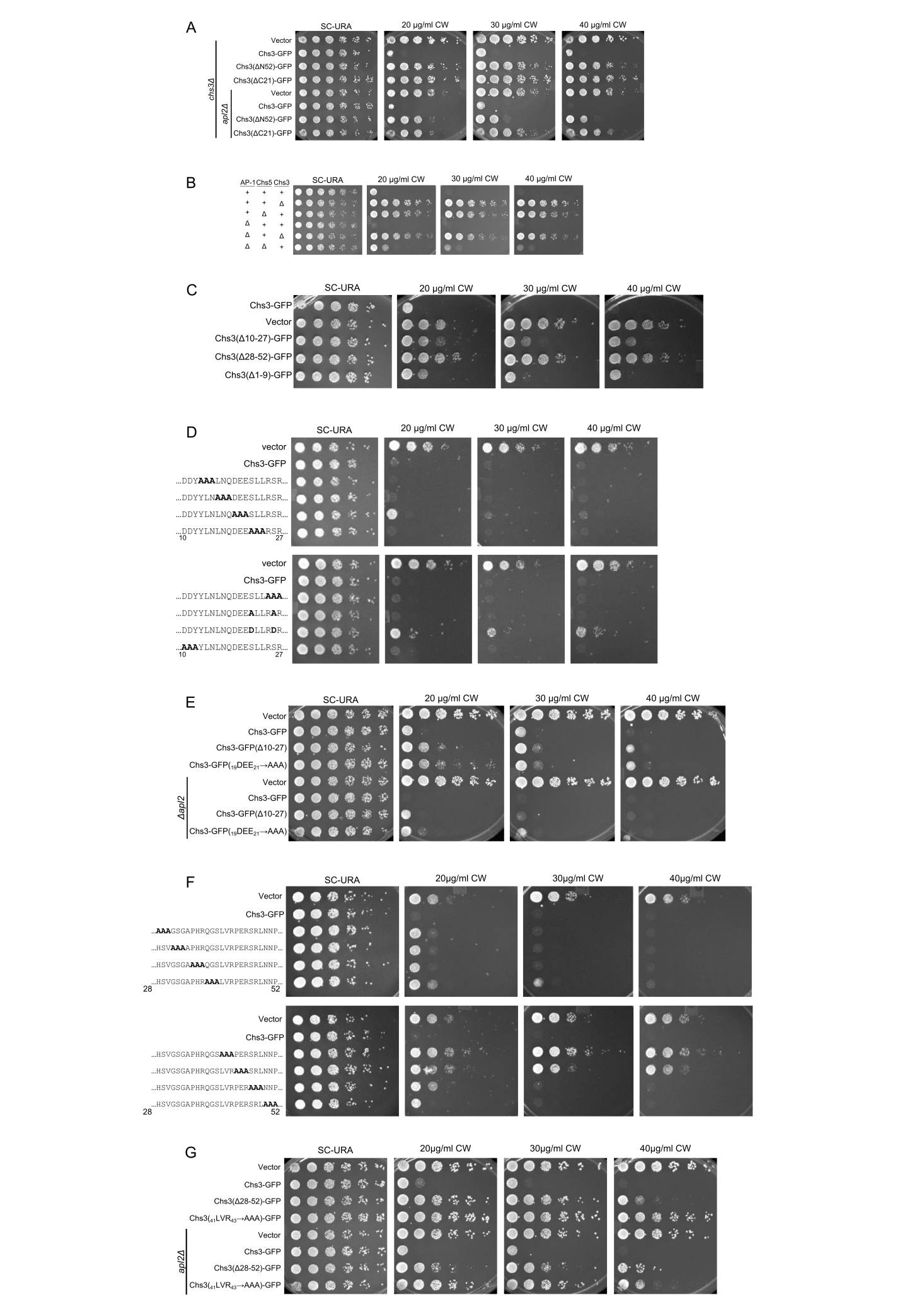


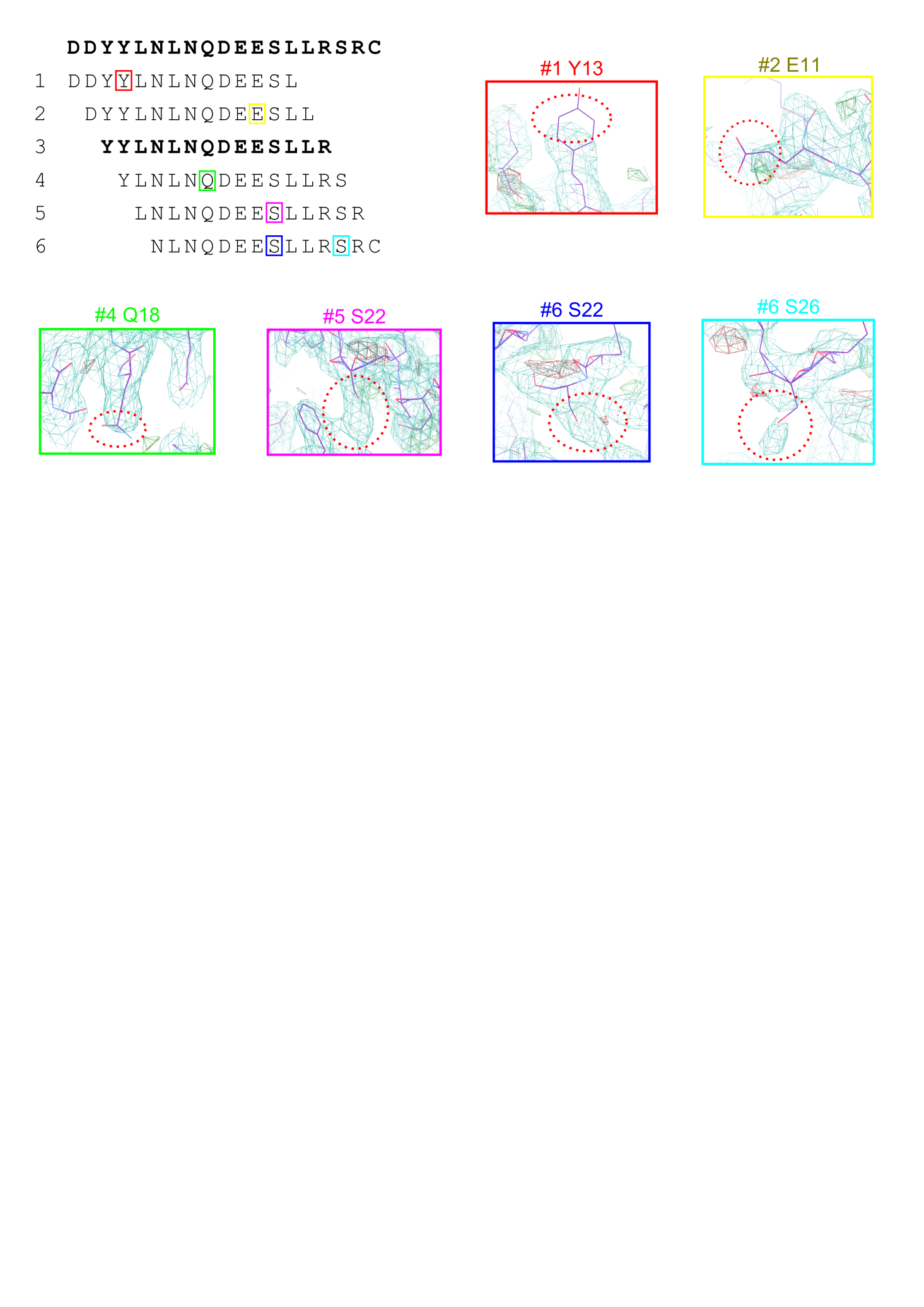
Supplementary Figure 2. Additional CW concentrations. Full range of calcofluor white concentrations tested for each of the experiments shown in the indicated main figures. (A) and (B): Figure 1. (C): Figure 2. (D) and (E): Figure 3. (F) and (G): Figure 4.

**Supplementary Figure 3. Determination of peptide register**. While the peptide used for co-crystallization contained 18 residues (including an added cysteine at the C-terminus), only 14 could be seen in the electron density, resulting in 6 possible registers (top left). Number 3 appeared to be the best fit. Images show side chains that are a poor fit for the electron density if the indicated register is used. Since this region contains so many large residues and the side chains facing away from the complex are poorly defined, the actual peptide register is uncertain.

1. **References**

Paczkowski, J. E., Richardson, B. C., Strassner, A. M., and Fromme, J. C. (2012). The exomer cargo adaptor structure reveals a novel GTPase-binding domain. *EMBO J.* 31, 4191–203. doi:10.1038/emboj.2012.268.

Richardson, B. C., and Fromme, J. C. (2013). The exomer cargo adaptor features a flexible hinge domain. *Structure* 21, 486–92. doi:10.1016/j.str.2013.01.003.

Robinson, J., and Klionsky, D. (1988). Protein sorting in Saccharomyces cerevisiae: isolation of mutants defective in the delivery and processing of multiple vacuolar hydrolases. *Mol. Cell. Biol.* doi:10.1128/MCB.8.11.4936.Updated.

Sikorski, R., and Hieter, P. (1989). A system of shuttle vectors and yeast host strains designed for efficient manipulation of DNA in Saccharomyces cerevisiae. *Genetics*.
